# The inhibition of checkpoint activation by telomeres does not involve exclusion of dimethylation of histone H4 lysine 20 (H4K20me2)

**DOI:** 10.12688/f1000research.15166.2

**Published:** 2018-10-09

**Authors:** Julien Audry, Jinyu Wang, Jessica R. Eisenstatt, Kathleen L. Berkner, Kurt W. Runge

**Affiliations:** 1 Department of Inflammation and Immunity, Lerner Research Institute, Cleveland Clinic, Lerner College of Medicine, Case Western Reserve University, Cleveland, OH, 44195, USA; 2 Department of Genetics and Genomic Sciences, School of Medicine, Case Western Reserve University, Cleveland, Ohio, 44106, USA; 3 Department of Biochemistry, School of Medicine, Case Western Reserve University, Cleveland, OH, 44106, USA; 4 Department of Molecular Cardiology, Lerner Research Institute, Cleveland Clinic, Lerner College of Medicine, Case Western Reserve University, Cleveland, OH, 44195, USA

**Keywords:** Fission yeast, H4K20me2, histone, methylation, DNA damage, checkpoint, telomere

## Abstract

DNA double-strand breaks (DSBs) activate the DNA damage checkpoint machinery to pause or halt the cell cycle.  Telomeres, the specific DNA-protein complexes at linear eukaryotic chromosome ends, are capped DSBs that do not activate DNA damage checkpoints.  This “checkpoint privileged” status of telomeres was previously investigated in the yeast *Schizosaccharomyces pombe*lacking the major double-stranded telomere DNA binding protein Taz1. Telomeric DNA repeats in cells lacking Taz1 are 10 times longer than normal and contain single-stranded DNA regions. DNA damage checkpoint proteins associate with these damaged telomeres, but the DNA damage checkpoint is not activated. This severing of the DNA damage checkpoint signaling pathway was reported to stem from exclusion of histone H4 lysine 20 dimethylation (H4K20me2) from telomeric nucleosomes in both wild type cells and cells lacking Taz1.  However, experiments to identify the mechanism of this exclusion failed, prompting our re-evaluation of H4K20me2 levels at telomeric chromatin.  In this short report, we used an extensive series of controls to identify an antibody specific for the H4K20me2 modification and show that the level of this modification is the same at telomeres and internal loci in both wild type cells and those lacking Taz1.  Consequently, telomeres must block activation of the DNA Damage Response by another mechanism that remains to be determined.

## Editorial Note

The link to dataset 2 in the version 1 paper was not provided due to an editorial error. Thus, we now include below the original dataset 2 for readers to see (labelled Dataset 2), alongside the revised dataset 2 provided for the version 2 article (labelled Dataset 3).

## Introduction

Genome instability is a potentially lethal event for a eukaryotic cell, and a mutational force for genetic diseases such as cancer. DNA double-strand breaks (DSBs) can drive genome instability and are sensed by the DNA damage checkpoint, a defined set of evolutionarily-conserved proteins that bind the DSB to signal a pause or arrest of the cell cycle ^1^ and recruit proteins to repair the DNA lesion ^2, 3^. Telomeres, the physical ends of linear eukaryotic chromosomes, are specialized DSBs that suppress DNA damage checkpoint activation by an unknown mechanism(s), even though telomeres are bound by many of the DNA damage checkpoint proteins that signal cell cycle arrest ^4^. Carneiro *et al.* ( *Nature* 467: 228-232) addressed this question using *Schizosaccharomyces pombe* cells that lack Taz1, the protein that binds to double-stranded telomere repeats ^5^. Telomeres in *taz1∆* cells have single-stranded DNA regions that are bound by checkpoint and DNA repair proteins, but cells do not arrest ^5, 6^. Immunofluorescence co-localization results from Carneiro *et al.* indicated that the ortholog of the human DNA damage checkpoint protein 53BP1 (Crb2) found at DSBs was not recruited to telomeres ^5^. Crb2 can bind to dimethylated lysine 20 of histone H4 (H4K20me2) in nucleosomes ^7^. Carneiro *et al.* presented data that H4K20me2 was depleted near telomeres in wild type and *taz1∆* cells, suggesting a mechanism for checkpoint suppression ^5^. Efforts to pursue this exciting result by ourselves and others failed. We therefore carefully re-evaluated the presence of H4K20me2 at different chromosomal loci, and found that H4K20me2 is not depleted near telomeres, indicating that checkpoint suppression occurs by some other mechanism(s).

## Methods

### Construction of the H4K20R strains

Wild type (yJRE20-1) and histone H4 lysine 20 mutant (yJRE21-1) strains were previously described ^8^ and were constructed as follows: The 5’ flanking region, the *H4* gene, and the 3’ flanking region of each histone *H4* gene were separately cloned into a pFA vector 5’ of the selection marker ( *hhf1* into pFA6a arg3MX6; *hhf2* into pFA6a his3MX6; *hhf3* into pFA6a ura4MX6). Approximately 500 base pairs 3’ to the initial fragment was cloned 3’ of the selection marker to the appropriate vector. The inclusive distance between the last A for the histone H4 TAA stop codon and the first G in the Asc I site from the pFA6a marker was 441 bp from H4.1 to *arg3 ^+^*, 707 bp from H4.2 to *his3 ^+^* and 464 bp from H4.3 to *ura4 ^+^* (plasmid maps are included in Supplementary File 1). Each construct was verified by restriction enzyme digestion and DNA sequencing of the fragments. Site-directed mutagenesis was used to mutate lysine 20 to arginine (H4K20R) at each gene copy; for wild type strains, the site was left unmutated. The resulting mutant constructs were verified by enzyme digestion and capillary dye terminator dideoxyDNA sequencing at ACGT (ACGT, Inc., Germantown, MD) to confirm the codon change corresponding to H4K20 (examples of aligned sequences are available in Supplementary File 2). Linearized fragments containing the 5’ fragment, selectable marker, and 3’ fragment were separately transformed into FY1645 ( *hhf1, h ^+^*) or FY1646 ( *hhf2* and *hhf3, h ^-^*) ^9^. Confirmation of integration was done by restriction digestion and DNA sequencing of the PCR product of the H4 gene. The strains with *hhf1* ( *h ^+^*) and *hhf3* ( *h ^-^*) marked and/or mutated were crossed to generate a strain in which *hhf2* is the only unmarked gene copy. The resulting *h ^+^* strain was then crossed with the *hhf2* ( *h ^-^*) marked strain to generate a strain in which all three loci of the histone H4 gene contain a selectable marker and are either wild type or mutated to arginine at lysine 20. Confirmation via digestion and DNA sequencing was performed after each cross. The H4K20R strain has been previously shown to be sensitive to DNA damaging agents ^8^. The strains and primers used during strain construction are available upon request.

### ChIP assay

The strains used are described in Table 1. For the control strains lacking H4K20 methylation created by transformation for these experiments, two (yJRE141) or three (JA008) independent transformants were independently assayed in parallel. Cells were grown at 32°C in 300 ml in EMMG + AHRULK (yJRE141-3 and yJRF141-6) or EMMG + AHRULK + G418 (All other cells. EMMG is described in Moreno *et al*. ^10^ and AHRULK + G418 contains 225 mg/l adenine, histidine, arginine, uracil, leucine, lysine and 200 mg/l G418 sulfate). Mid-log cells (9–12 × 10 ^6^/ml or 0.8-1.2 OD _600_) were cross-linked with 1% formaldehyde for 15 min at room temperature and washed twice with cold HBS buffer (50 mM HEPES-NaOH pH 7.5, 140 mM NaCl). Cell pellets were stored at -80°C. All subsequent steps were performed at 4°C. Cell pellets were resuspended in ChIP-lysis buffer ^11^ and lysed using mechanical disruption by bead-beater (Bio Spec Mini-Beadbeater-16) with 0.5 mm glass beads (Biospec 11079105) using 4 cycles of 45 sec followed by 60 sec on ice. The lysate was sonicated for 10 cycles on maximum power (30 sec ON and 59 sec OFF) in a Diagenode Bioruptor XL with sample tubes soaked in an ice water bath. Solubilized chromatin protein (2–4 mg) was used for each ChIP while 5 µl was saved as Input. Antibodies (2 µg) against H4K20me2 (GeneTex GT282 [RRID: AB_2728656] Lot #41582) or total histone H4 (Abcam ab10158 [RRID: AB_296888] Lot #GR133660-1) were mixed with chromatin and incubated at 4°C while rocking for 4 h. Dynabeads Protein G (50 µl, Life Technologies, Cat. No. REF 10004D) was then added and rocked overnight at 4°C. Beads were washed with ChIP lysis buffer, ChIP lysis buffer with 500 mM NaCl, Wash buffer and TE buffer (10 mM Tris, 1 mM EDTA pH 7.5) successively ^11^. Beads were then resuspended in 145 µl of TES (1X TE with 1% SDS). Supernatant (120 µl) was recovered and incubated in a Thermomixer at 65°C, 1000 rpm (rotation per min) overnight to reverse cross-linking. For Input samples, TES buffer (115 µl) was added and incubated in the Thermomixer with the ChIP samples. Samples were treated with RNase A (2 µl of 10mg/ml added to each sample)(Roche 10109142001) for 15 min at 37°C and Proteinase K (2 µl of 20mg/ml added to each sample)(Roche 03115879001) for 30 min at 55°C, and purified by QIAgen PCR purification column (Cat.No. 28106) ^14^. All samples from the same assay were processed for ChIP assay at the same time.

**Table 1.** Yeast strains used in this study.

Name	Genotype	Source
yJRE20-1	*h ^-^ H4.1::arg3 ^+^ H4.2::his3 ^+^ H4.3::ura4 ^+^ ade6-210 arg3∆-4 his3∆-1 leu1-32* *ura4-D18*	This lab ^8^, used for western as *WT*
yJRE21-1	*h ^-^ H4.1-K20R::arg3 ^+^ H4.2-K20R::his3 ^+^ H4.3-K20R::ura4 ^+^ ade6-210 arg3∆-4* *his3∆-1 leu1-32 ura4-D18*	This lab ^8^, used for western as *H4K20R*
ySLS298	*h ^-^ set9::CYC-terminator-kanMX* ( *set9 ^+^* strain)	Greeson *et al*. ^12^, used for western as *set9-kan-wt*
ySLS252	*h ^-^ set9∆::CYC-terminator -kanMX* ( *set9*-deletion strain)	Greeson *et al*. ^12^, used for western as *set9∆*
yNTG41	*h ^-^ set9-F178Y::CYC-terminator -kanMX*	Greeson *et al*. ^12^, used for western as *set9-F178Y*
yNTG39	*h ^-^ set9-F164Y::CYC-terminator -kanMX*	Greeson *et al*. ^12^, used for western as *set9-F164Y*
yNTG43	*h ^-^ set9-F195Y::CYC-terminator -kanMX*	Greeson *et al*. ^12^, used for western as *set9-F195Y*
yJRE210-1	*h ^+^ ade6-210 arg3-D4 his3-D1 leu1-32::pFA-LEU2-I-SceI ura4-D18 gal1-3'::* *ura4 ^+^-48bp TeloRpt-I-SceI-hph ^+^*	This lab ^13^, used for ChIP as wild type
JA002-3	*taz1∆::kanMX* introduced into yJRE210-1 by transformation	This work, used for ChIP as *taz1∆*
JA008-1	*set9∆::kanMX* introduced into yJRE210-1 by transformation	This work, used for ChIP as *set9∆*
JA008-2	*set9∆::kanMX* in yJRE210-1, independent transformant from JA008-1	This work, used for ChIP as *set9∆*
JA008-3	*set9∆::kanMX* in yJRE210-1, independent transformant from JA008-1 and JA008-2	This work, used for ChIP as *set9∆*
yJRE141-3	*h ^-^ ade6-210 arg3-D4 his3-D1 leu1-32 ura4-D18 hhf1K20R::arg3 ^+^ hhf2K20R::* *his3 ^+^ hhf3K20R::nat ^R^ leu1-32::pFA-LEU2-TETp-I-SceI gal1-3’::ura4 ^+^-48 bp* *TeloRpt-I-SceI-hph ^+^*	This lab ^8^, used for ChIP as H4K20R
yJRE141-6	Independent isolate of yJRE141-3	This lab ^8^, used for ChIP as H4K20R

### qPCR Analysis for ChIP

Input samples were diluted to 1/100 with ddH _2_O while beads-only-ChIP, H4-ChIP and H4K20me2-ChIP samples were diluted to 1/10. Template DNAs (4 µl) were added to 5 µl of Roche LightCycler 480 SYBR Green I Master (2X) and primers (final concentration 0.6 µM) for a 10 µl total reaction volume. Each sample was run in triplicate on the same 384-well PCR plate (Roche LightCycler 480 Multiwell Plate 384, clear) in a Roche LightCycler 480. H4K20me2 immunoprecipitate levels were normalized to the total H4 immunoprecipitate levels at each locus ^15– 17^. The primers used are shown in Table 2, all primers were custom syntheses purchased from Integrated DNA Technologies (Skokie, IL, USA). Each locus was assayed using two or three primer pairs in the same qPCR assay for each ChIP, and the results for all primer pairs for a specific locus were averaged to obtain the final ChIP signal. The level of H4K20me2 at each locus was calculated as a ratio of H4K20me2 ChIP level to H4 ChIP level, where each ChIP level is expressed as a percent of input chromatin in the immunoprecipitated DNA (i.e. amount of DNA in H4K20me2 ChIP H4K20me2/amount DNA in the input chromatin divided by amount of DNA in H4 ChIP/amount DNA in the input chromatin).

**Table 2.** Primers used for qPCRs for ChIP. All primers were custom syntheses purchased from IDTdna.com.

Name	Sequence	Reference
79 act1 1-1Fw	TGC CGA TCG TAT GCA AAA GG	Oya *et al*., 2013 ^18^
80 act1-1Rev	CCG CTC TCA TAC TCT TG	Oya *et al*., 2013 ^18^
139 act1-2Fw	GCA AGC GTG GTA TTT TGA CC	This study
140 act1 2Rev	TCA GTC AAC AAG CAA GGG TG	This study
141 act1-3Fw	TAC CAC TGG TAT CGT CTT GG	This study
142 act1-3Rev	TAG TCA GTC AAG TCA CGA CC	This study
143 hip3-1Fw	AGC CAA ATT TGA CGG TGT TC	This study
144 hip3-1Rev	AGA CCT GGA CGG CAT TTT TA	This study
145 hip3-2Fw	GGT GCC AAG ATT GTT TAT CCA	This study
146 hip3-2Rev	ACG ACG TAT CCG ACA TCC TC	This study
147 hip3-3Fw	ACG ATG CCG AGT AGT TCA GC	This study
148 hip3-3Rev	TTC GTT GTT GTG TGC CTT TC	This study
135 Telo-1Fw	CAG TAG TGC AGT GTA TTA TGA TAA AAA TGG	Carneiro *et al*., 2010 ^5^
136 Telo-1Rev	CAG TAG TGC AGT GTA TTA TGA TAA TTA AAA TGG	Carneiro *et al*., 2010 ^5^
121 Telo-2Fw	TAT TTC TTT ATT CAA CTT ACC GCA CTT C	Kanoh *et al*., 2005 ^19^
122 Telo-2Rev	CAG TAG TGC AGT GTA TTA TGA TAA TTA AAA TGG	Kanoh *et al*., 2005 ^19^

### Cell extract preparation

Cells of 5 OD (5 × 10 ^7^ cells) were collected and resuspended in 200 µl SDS loading buffer without dye and reducing agent (50 mM Tris, 2% SDS, 10% glycerol). Cells were lysed using mechanical disruption by FastPrep 120 (Thermo Savant) with 0.5 mm glass beads, in cold room, using 2 cycles of 40 sec of disruption followed by 60 sec on ice. Cell lysis efficiency, monitored under microscope, always reached a minimum of 90%. The lysate was collected by poking holes on the bottom of the tubes and spinning into new tubes at 1000 rpm for 1 min at 4°C. The protein concentration was measured by BCA assay (Pierce 23225) on 96-well plate. After adding 4X SDS loading buffer, lysate of 10 µg was heated at 95°C for 5 min, spun down, and loaded into each lane on SDS-PAGE gel. The rest of the lysates were kept at -20°C.

### Recombinant histone H4 preparation

Recombinant histone H4 (MLA-modified) H4K20me1 or H4K20me2 or H4K20me3 (Active Motif® 31224, 31225, 31226) and unmodified recombinant histone H4 (Active Motif® 31223) were resuspended in PBS buffer (in HPLC grade water) and used at the working concentration of 50 ng/μl except for H4K20me3 which was at 2.5 ng/μl. After adding 4X SDS loading buffer, 200 ng of recombinant histone H4 was heated at 95°C for 5 min, spun down, and loaded into each lane on SDS-PAGE gel. The rest of the proteins were stored at -20°C.

### Western analysis

SDS-PAGE gels were prepared with a 15% resolving gel and a 4% stacking gel using 40% Acrylamide/Bis solution (BioRad 161-0146), Tris buffer and SDS. The gel was run in 1x SDS-Glycine-Tris running buffer with Odyssey One-Color Molecular Weight Protein Marker (Li-Cor 928-40000). The proteins were transferred onto nitrocellulose membrane (Li-Cor 926-31092) using Genie transfer system for 1 h with 1X transfer buffer with 20% methanol and 0.05% SDS. The membrane was stained with Ponceau S and the blot above the 25 kDa marker band was removed. The cut membrane was then rocked with Odyssey blocking buffer (Li-Cor 927-40000) at room temperature for 1 h, followed by incubation with anti-H4K20me2 antibodies (GeneTex GT282 [RRID: AB_2728656] Lot #41582) diluted 1:2000 in Odyssey blocking buffer with 0.2% Tween-20 at 4°C overnight. In some experiments, GT282 was replaced with Abcam ab9052 ([RRID:AB_1951942] lot #GR99672-1). Anti-H4 antibody (Abcam ab10158 [RRID: AB_296888] Lot #GR133660-1) was diluted at 1:10000 in Odyssey blocking buffer with 0.2% Tween-20. The anti-H4K20me2 blot was treated with the secondary antibody 680RD Goat anti-Mouse IgG (Li-Cor 926-68070 [RRID: AB_10956588]) in Odyssey blocking buffer with 0.2% Tween-20 at room temperature and rocked for 1 h and kept away from light during the incubation. For anti-H4 blots, the secondary antibody was Goat anti-Rabbit antibody IgG (800CW Li-Cor 926-32211 [RRID: AB_621843]). The blots were scanned by the Odyssey® CLx Imaging system to acquire Western blot signal and analyzed with the Image Studio ^TM^ software (v. 4.0).

unedited blot images Click here for additional data file. Copyright: © 2018 Audry J et al. 2018 Data associated with the article are available under the terms of the Creative Commons Zero "No rights reserved" data waiver (CC0 1.0 Public domain dedication).

Original excel workbook containing the Ct values from the PCRs and the location of the primers within the genes and telomere repeat adjacent DNA Click here for additional data file. Copyright: © 2018 Audry J et al. 2018 Data associated with the article are available under the terms of the Creative Commons Zero "No rights reserved" data waiver (CC0 1.0 Public domain dedication).

Revised excel workbook containing the Ct values from the PCRs, the location of the primers within the genes and telomere repeat adjacent DNA and the ChIP values of total H4 normalized to input chromatin Click here for additional data file. Copyright: © 2018 Audry J et al. 2018 Data associated with the article are available under the terms of the Creative Commons Zero "No rights reserved" data waiver (CC0 1.0 Public domain dedication).

## Results and discussion

**Figure 1.**
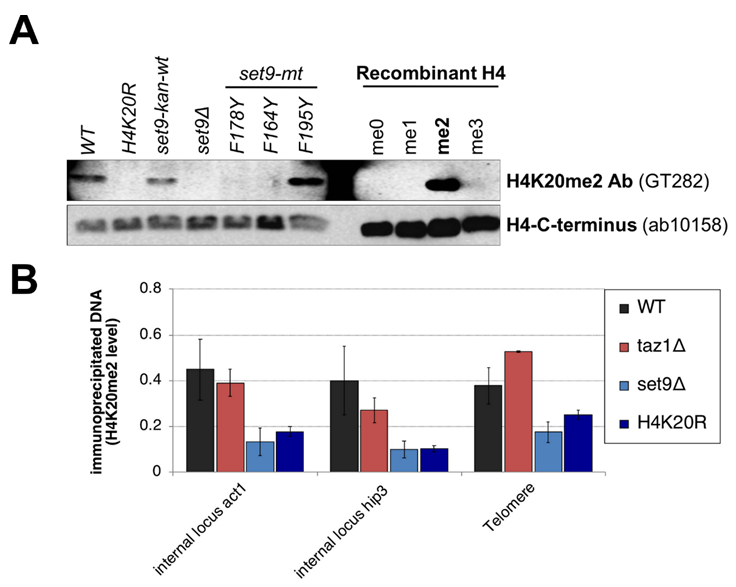
*H4K20me2* is not excluded from the telomere repeat-adjacent nucleosomes in wild type or *taz1∆* cells. An antibody that specifically recognizes H4K20me2 was identified ( **A**) and used in ChIP to measure levels of H4K20me2 in chromatin at two standard internal loci and loci adjacent to the telomere repeats ( **B**). H4K20me2 levels are expressed as the ratio of the H4K20me2 ChIP levels (% of DNA in anti-H4K20me2 IP compared to input chromatin) over H4 ChIP levels (% of DNA in anti-histone H4 IP compared to input chromatin). Wild type and *taz1∆* cells have the same levels at all loci and are clearly distinguishable from the negative controls ( *P* values compared to wild type levels: all *taz1∆* strains >0.18; all *set9∆* and H4K20R strains ≤0.023, individual values are presented in Table 3). Each western blot in panel **A** used a separate, identically run gel to analyze the samples shown. M stands for molecular weight markers.

If the DNA damage checkpoint at telomeres is severed by excluding H4K20me2 from telomeric chromatin, the presence of H4K20me2 in telomeric nucleosomes would activate the DNA damage response in *taz1∆* cells, causing slower growth or cell cycle arrest. H4K20me2 constitutes ~25% of total H4 in *S. pombe* ^20^, implicating a telomere-associated demethylase to deplete H4K20me2 at telomeres in *taz1∆* cells to prevent checkpoint-mediated arrest. However, both a genome-wide screen of gene deletion mutants (D. Durocher, pers. comm.) and our screen of demethylase mutants failed to identify a mutant that caused *taz1∆* cells to arrest, to grow poorly or to recruit more Crb2. We therefore re-evaluated H4K20me2 levels by chromatin immunoprecipitation (ChIP). We first identified commercial antibodies specific for H4K20me2 by western analysis using 11 different samples. Positive controls were extracts from wild type cells, cells where the single *S. pombe* H4K20 methylase gene *set9* is marked and functional ( *set9-kan-wt*) and recombinant H4 where the only modification is a chemical mimetic for K20me2 ^21^. Negative controls included recombinant H4 where the only modifications were mimetics of 0, 1 or 3 methyl groups on lysine 20, and extracts from cells where all three copies of the H4 gene have lysine 20 mutated to arginine (H4K20R) ^8^. A series of *set9* mutants that methylate H4K20 to contain 0 ( *set9∆*), 1 ( *set9-F164Y, set9-F178Y*), or 1 and 2 methyl groups ( *set9-F195Y*) were also assayed ^12^. The specific antibody identified ( Figure 1A) was used in ChIP to monitor H4K20me2 at the telomeric loci assayed in Carneiro *et al.* and two internal chromosomal loci in wild type and *taz1∆* cells, and in mutants that lack H4K20 methylation, *set9∆* and H4K20R. We also tested the antibody Carneiro *et al.* reported using to detect H4K20me2, Abcam ab9052. We found that this lot of ab9052 did not recapitulate the reactivity of the original antibody reported by Greeson ^12^ for different *set9* mutants and showed reduced reactivity to H4K20me2 ( Supplementary Figure 1) compared to the GT282 antibody ( Figure 1A), so this antibody was not used. Total H4 levels at these loci were monitored with an antibody that recognizes all H4 forms.

**Table 3.** *P* values for H4K20me2 levels compared to wild type in Figure 1. Assays were performed as described in Materials and Methods with two or more independent ChIP experiments. Each ChIP experiment was analyzed in triplicate. *P* values are from t-tests comparing each locus in a mutant strain to the same locus in the wild type strain, where values <0.05 are considered significant.

Strain	Locus	*P*
*taz1∆*	Internal locus *act1*	0.8913
*taz1∆*	Internal locus *hip3*	0.3377
*taz1∆*	Telomere: adjacent to telomere repeats	0.1842
*set9∆*	Internal locus *act1*	0.0082
*set9∆*	Internal locus *hip3*	0.0137
*set9∆*	Telomere: adjacent to telomere repeats	0.0213
H4K20R	Internal locus *act1*	0.0230
H4K20R	Internal locus *hip3*	0.0161
H4K20R	Telomere: adjacent to telomere repeats	0.0220

We found that H4K20me2 levels are similar at telomeres and the two internal loci in wild type and *taz1∆* cells, and clearly distinguishable from the *set9∆* and H4K20R negative controls ( Figure 1B). These results were obtained by normalizing H4K20me2 signals to total H4 signals, which allows the direct comparison of this H4 modification at loci which contain nucleosomes. Carneiro *et al.* normalized to their ChIP signals to total input chromatin ^5^. However, because telomere repeats are bound by non-nucleosomal proteins ^22^, this normalization gives a much lower ChIP signal for total H4 and, thus, a lower signal for all H4 modifications. An example of this lower H4 signal is shown in the third spreadsheet of Dataset 3, where the level of H4 at wild type telomeres is 1/5 to 1/9 that of internal loci. Therefore, normalization of H4K20me2 ChIP signals to total H4 is necessary to monitor the fraction of modified H4 at telomeres.

The results in Figure 1B argue that while the damaged telomeres in *taz1∆* cells block checkpoint activation, the mechanism is unlikely to be the suppression of H4K20me2 in telomeric chromatin. These results and conclusion are consistent with the genetic screen results that did not identify a demethylase required to sever the checkpoint in *taz1∆* cells and suggest that searches for combinations of demethylase mutants that activate the checkpoint in *taz1∆* cells will not be fruitful. Rather, broader approaches to investigate the differences between telomeres and DSBs may be required, including much more extensive characterization of the post-translation modifications of proteins at or near telomeres. While H4K20me2 levels are not reduced at telomeres, it is worth noting that checkpoint activation is the sum of multiple protein interactions and modifications, e.g. phosphorylation of histone H2A and modification of several checkpoint proteins ^23, 24^. Reducing the efficiency of some of these interactions may be sufficient to impair checkpoint signaling at *taz1∆* cell telomeres. Results from such studies may provide an understanding of the anti-checkpoint activity of telomeres so that it may be modulated to treat telomere-related diseases such as cellular aging and cancer ^25^.

## bioRxiv

A previous version of this article is available from bioRxiv - https://doi.org/10.1101/251389 ^26^

## Data availability

The data referenced by this article are under copyright with the following copyright statement: Copyright: © 2018 Audry J et al.

Data associated with the article are available under the terms of the Creative Commons Zero "No rights reserved" data waiver (CC0 1.0 Public domain dedication). 

Dataset 1: unedited blot images 10.5256/f1000research.15166.d209374 ^27^

Dataset 2: Original excel workbook containing the Ct values from the PCRs and the location of the primers within the genes and telomere repeat adjacent DNA. 10.5256/f1000research.15166.d220802 ^28^

Dataset 3: Revised excel workbook containing the Ct values from the PCRs, the location of the primers within the genes and telomere repeat adjacent DNA and the ChIP values of total H4 normalized to input chromatin. 10.5256/f1000research.15166.d219736 ^29^

## Supplementary material

Supplementary File 1: Plasmid maps. Files are in Snapgene Reader format, a free software package available here: http://www.snapgene.com/products/snapgene_viewer/

Supplementary File 2: Examples of plasmid sequences used to validate mutation

Supplemental Figure 1. Western blot results with the ab9052 antibody.
