# Supplementary material for: The inhibition of checkpoint activation by telomeres does not involve exclusion of dimethylation of histone H4 lysine 20 (H4K20me2)
Source: F1000Res. 2018 Oct 9;7:1027. Originally published 2018 Jul 9. [Version 2] doi: 10.12688/f1000research.15166.2 (PMC6240467; doi:10.12688/f1000research.15166.2)
Supplement: Supplementary file 5 [file f1000research-7-17976-s0004.tgz › b14e9630-33e9-4fca-9b81-b8375198db22.pptx]

## Slide 1
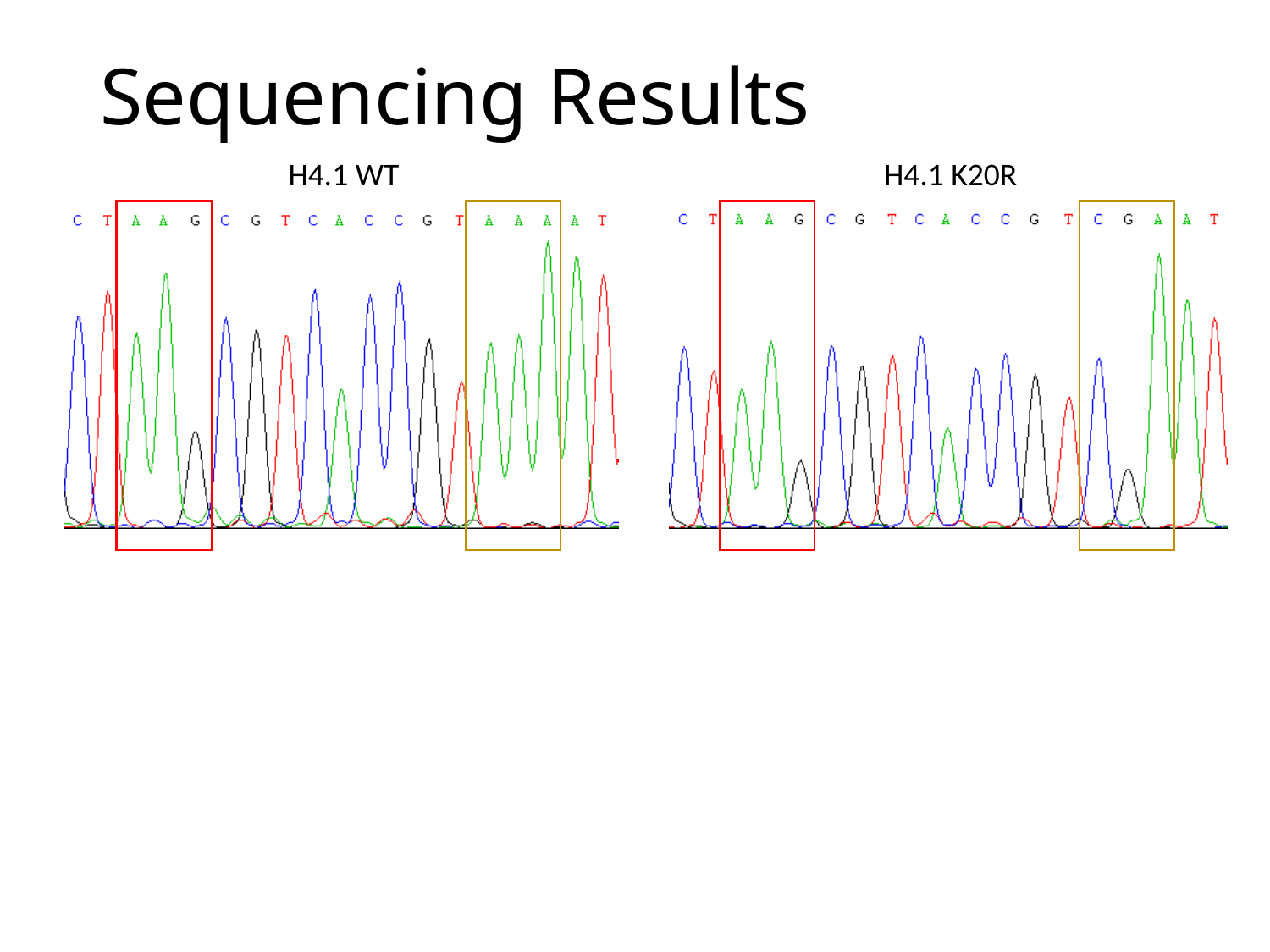

# Sequencing Results
H4.1 WT
H4.1 K20R

## Slide 2
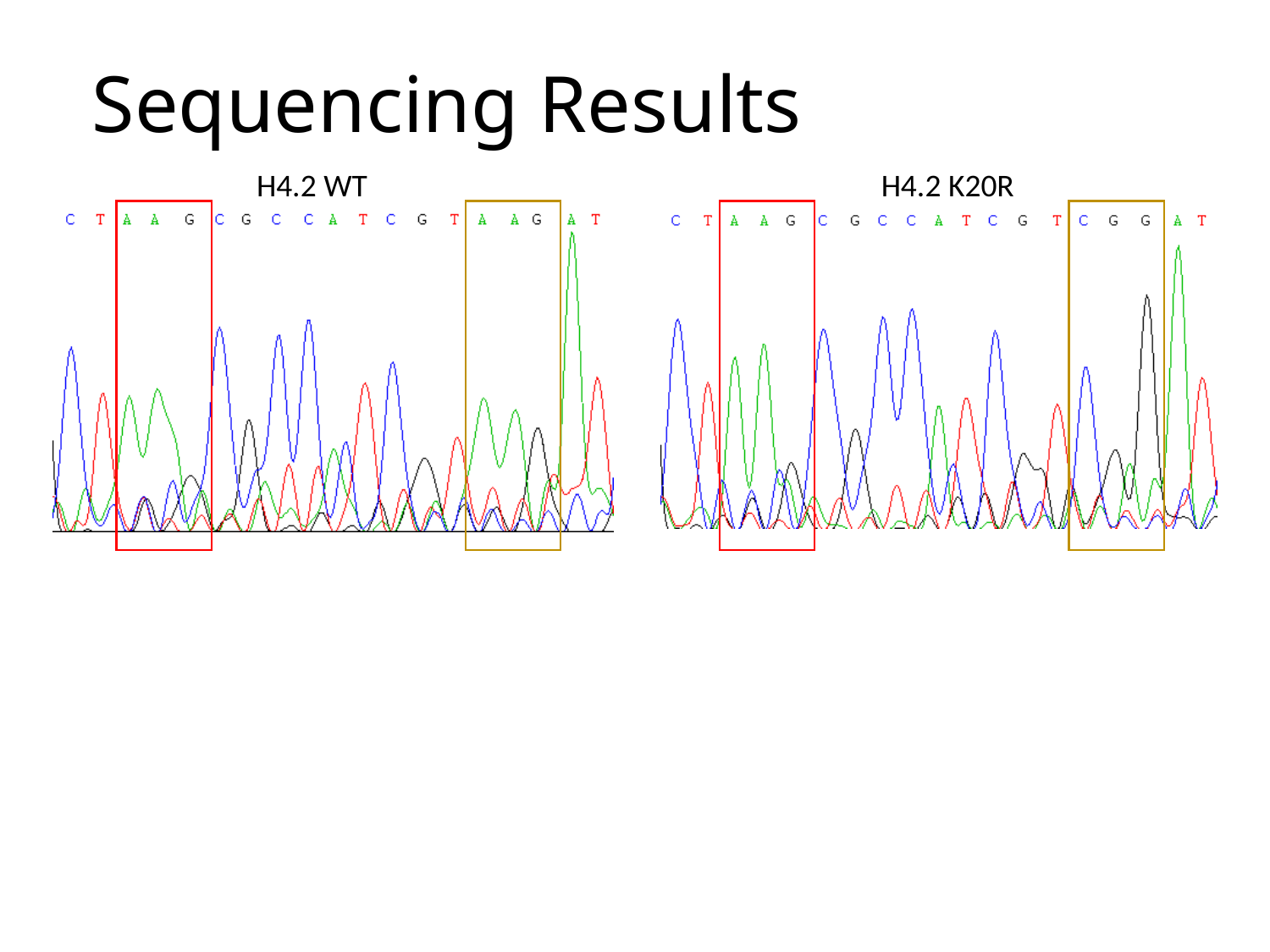

# Sequencing Results
H4.2 WT
H4.2 K20R

## Slide 3
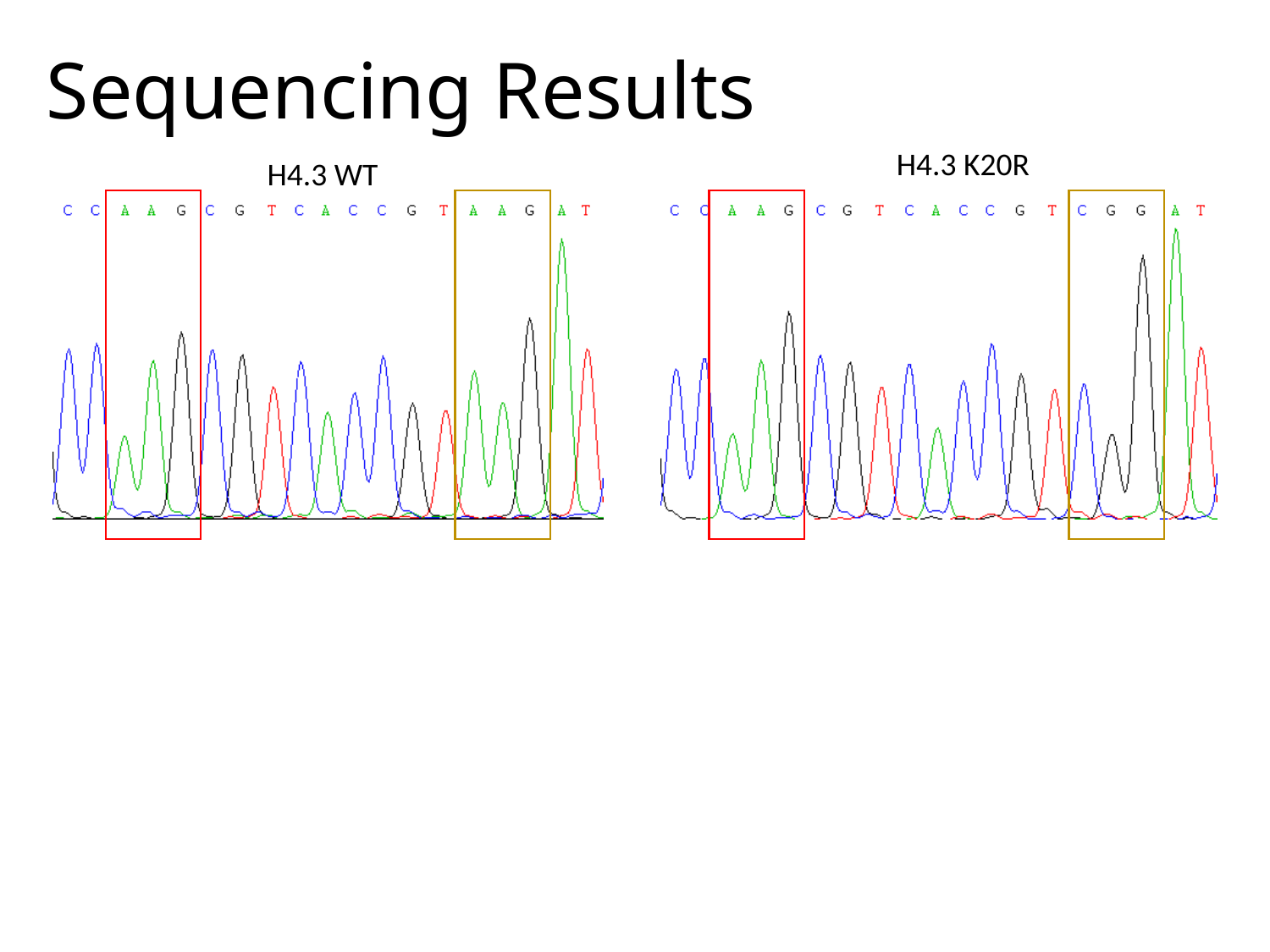

# Sequencing Results
H4.3 K20R
H4.3 WT
