# Supplementary material for: The inhibition of checkpoint activation by telomeres does not involve exclusion of dimethylation of histone H4 lysine 20 (H4K20me2)
Source: F1000Res. 2018 Oct 9;7:1027. Originally published 2018 Jul 9. [Version 2] doi: 10.12688/f1000research.15166.2 (PMC6240467; doi:10.12688/f1000research.15166.2)
Supplement: Supplementary file 6 [file f1000research-7-17976-s0005.tgz › 238660f2-f9b2-48e7-9e42-aebfa7fb9bef_Supplementary_Figure_1.docx]

**
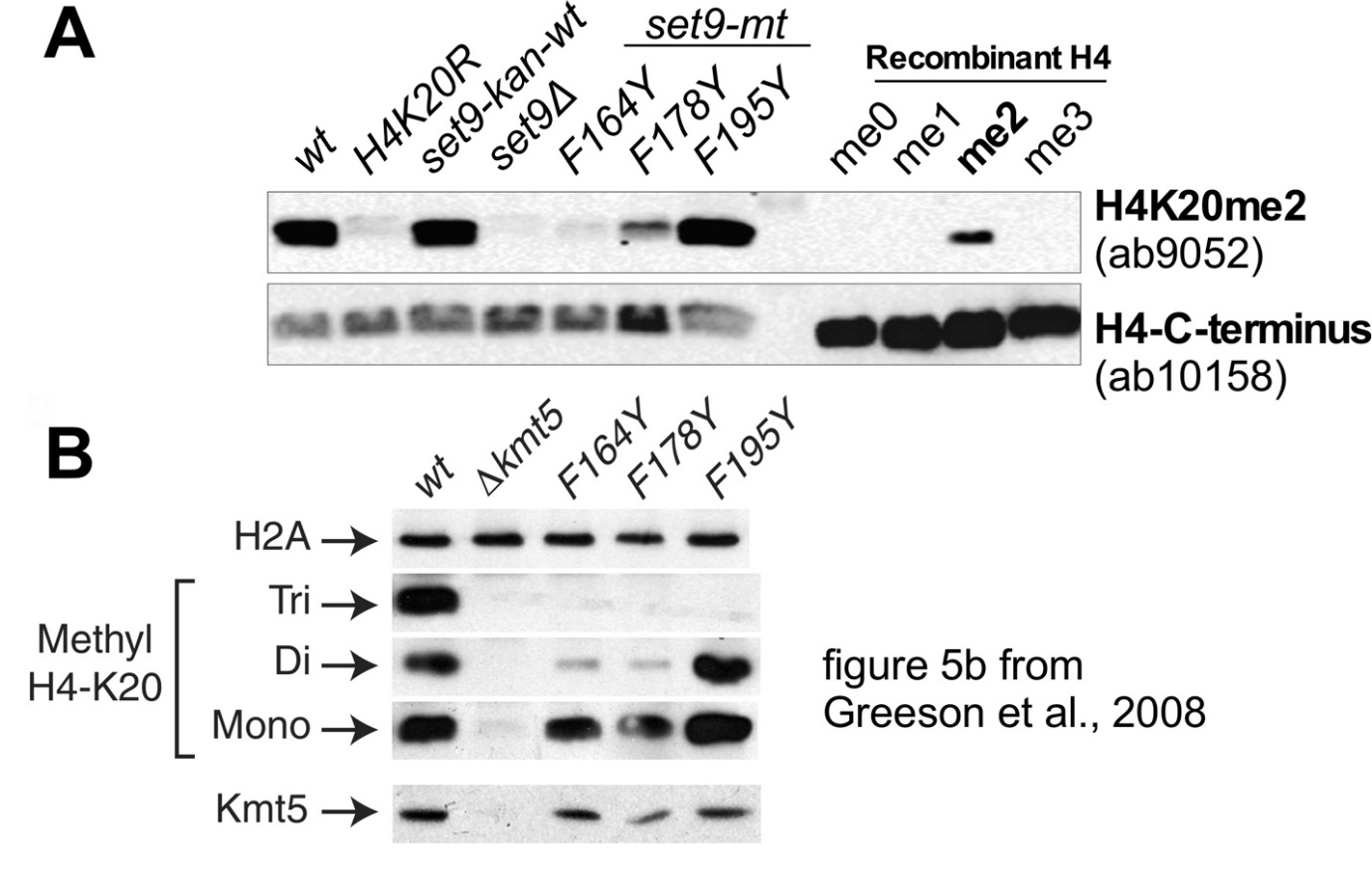
**

**Supplementary Figure 1. Western blot results with the ab9052 antibody.**

The affinity of the Abcam ab9052 anti-H4K20me2 antibody (lot #GR99672-1) appears altered compared to the original antibody. A) The test of Abcam antibody ab9052 for H4K20me2 performed as in Figure 1. The ab9052 antibody shows reduced reactivity with the recombinant H4K20me2 and shows increased reactivity with the histones in from the *set9-F178Y* mutant compared to the GT282 antibody in Figure 1. The GT282 antibody was therefore used in the ChIP experiments. B) Figure 5b from Greeson *et al.* 2008, performed with the original Abcam ab9052 antibody using protein extracts from the same *set9* mutants shown in panel A. The differences between the blot from Figure 5b in Greeson *et al*. and the blot in panel A raised concerns that the most recent lot of ab9052 would not provide adequate specificity for ChIP.
