# Supplementary material for: The inhibition of checkpoint activation by telomeres does not involve exclusion of dimethylation of histone H4 lysine 20 (H4K20me2)
Source: F1000Res. 2018 Oct 9;7:1027. Originally published 2018 Jul 9. [Version 2] doi: 10.12688/f1000research.15166.2 (PMC6240467; doi:10.12688/f1000research.15166.2)
Supplement: Peer review history

# Peer review: Referee response for version 2

**DOI:** 10.5256/f1000research.17976.r39273

**Published:** 2018-10-15

**Authors:** Jeffrey S. Thompson

**Affiliations:** 1 Department of Biology, Denison University, Granville, OH, USA

Audry et al. examine a previously published observation (Carneiro et al., 2010 ^1^) that checkpoint activation is inhibited at telomeres as a result of exclusion of histone H4K20me2 at these loci. The prior observation was done via ChIP, using a polyclonal antibody from Abcam. As addressed in the present manuscript, the inability to identify a demethylase that is associated with this effect raised questions as to the certainty of the claim. The investigators of the current study have identified what appears to be a superior antibody to evaluate this modification. The antibody from GeneTex shows improved specificity for the dimethylated state relative to that observed for the previously utilized antibody from Abcam, and furthermore displays higher affinity for H3K20me2 relative to the currently available lot of the Abcam antibody. 

 The current study clearly demonstrates that H4K20me2 is detectable at telomeric loci, and that no differences are observed in taz1 mutant strains. The investigators took steps to normalize the ChIP data relative to total H4 levels to ensure that loci-specific variations in nucleosome levels were accounted for, which is completely appropriate for telomeric regions where histone levels are reduced relative to internal loci. Thus, the findings presented here raise further doubts about the role of H4K20me2 in checkpoint inhibition at telomeres.

 This study provides useful insights into the nature of DNA damage checkpoint activation, and the underlying mechanisms by which cells inhibit such pathways in response to native chromosomal features such as telomeres. While the original finding, implicating reduced H4K20me2 levels at telomeres as an inhibitor of checkpoint activation, was supported by the previously published observations, it is essential that such models adapt as new information is made available. The use of antibodies for gauging histone modification levels has been perpetually problematic for many years, as these reagents are routinely used to evaluate subtle molecular changes across large genomic landscapes, pushing the limits of the methodology. In particular, lot variation for polyclonal antibodies is a serious problem in this field. In the absence of more reliable methodologies, we must be willing to reconsider prior observations as new antibodies become available. The study presented here appropriately re-examines this prior observation with an improved reagent for detecting this modification, and the observations raise reasonable doubts regarding the original model. This study by no means settles the issue of the role (or lack thereof) of H4K20me2 in checkpoint inhibition, but the findings merit publication to ensure that other investigators continue to re-examine this model.

I have read this submission. I believe that I have an appropriate level of expertise to confirm that it is of an acceptable scientific standard.

## References

[1] CarneiroTKhairLReisCCBorgesVMoserBANakamuraTMFerreiraMG: Telomeres avoid end detection by severing the checkpoint signal transduction pathway. *Nature*.2010;467(7312) : 10.1038/nature09353 228-32 10.1038/nature09353 20829797PMC3196630

# Peer review: Referee response for version 1

**DOI:** 10.5256/f1000research.16521.r35871

**Published:** 2018-08-07

**Authors:** Holger Richly

**Affiliations:** 1 Laboratory of Molecular Epigenetics, Institute of Molecular Biology (IMB), Mainz, Germany

The DNA damage response involves the setting of specific chromatin marks that are important to recruit or maintain DNA repair factors at the damage site. One such mark is H4K20me2, which has been linked to doublestrand break repair (DSB repair) (Fradet-Turcotte 2013 ^1^) and more recently to nucleotide excision repair (NER) (Chitale et al 2018 ^2^). H4K20me2 is read by 53BP1 or Crb1, respectivley, which bridges the interaction with other DNA repair proteins thereby playing an essential role in chromatin signaling events at the damage site. Telomeres can be considered specialized DSBs in that they suppress the DNA damage checkpoint activation. One potential mechanism to explain this phenomenon was put forward by Carneiro and colleagues ^3^. In their research paper it was reported that 53BP1 (Crb2) and H4K20me2 do not locate to telomeres, which would partially explain why the DNA damage checkpoint activation was suppressed. However, this exciting finding was never recapitulated by other research groups. 

 Here, Audry and colleagues carefully reassessed the occurence of H4K20me2 at telomeres. To this end H4K20 mutant *S. pombe*strains were generated and most of the comercially available antibodies specifically recognizing the H4K20me2 mark were tested. Importantly, the authors found an antibody that shows the desired specificity when probing it against recombinant proteins and protein extracts from mutant strains. Employing this highly specific antibody in ChIP experiments they can unequivocally show that telomeres are decorated with H4K20me2, even in taz1 knockout cells, which is in stark contrast to the previous findings by Carneiro and colleagues. Thus, the findings presented here refute the idea that the DNA damage checkpoint is suppressed by exclusion of H4K20me2. This important finding will create a profound repercussion for the research field of genome stability as it shows that the mechanisms for checkpoint suppression at telomers have yet to be uncovered.

I have read this submission. I believe that I have an appropriate level of expertise to confirm that it is of an acceptable scientific standard.

## References

[1] Fradet-TurcotteACannyMDEscribano-DíazCOrthweinALeungCCHuangHLandryMCKitevski-LeBlancJNoordermeerSMSicheriFDurocherD: 53BP1 is a reader of the DNA-damage-induced H2A Lys 15 ubiquitin mark. *Nature*.2013;499(7456) : 10.1038/nature12318 50-4 10.1038/nature12318 23760478PMC3955401

[2] ChitaleSRichlyH: DICER- and MMSET-catalyzed H4K20me2 recruits the nucleotide excision repair factor XPA to DNA damage sites. *J Cell Biol*.2018;217(2) : 10.1083/jcb.201704028 527-540 10.1083/jcb.201704028 29233865PMC5800799

[3] CarneiroTKhairLReisCCBorgesVMoserBANakamuraTMFerreiraMG: Telomeres avoid end detection by severing the checkpoint signal transduction pathway. *Nature*.2010;467(7312) : 10.1038/nature09353 228-32 10.1038/nature09353 20829797PMC3196630

# Peer review: Referee response for version 1

**DOI:** 10.5256/f1000research.16521.r36001

**Published:** 2018-07-17

**Authors:** Miguel Godinho Ferreira, Toru M. Nakamura

**Affiliations:** 1 Instituto Gulbenkian de Ciência, Lisbon, Portugal; 2 Department of Biochemistry and Molecular Genetics, University of Illinois at Chicago, Chicago, IL, USA

Carneiro et al. 2010 described a mechanism whereby telomeres can prevent cell cycle arrest even when Rad3-Rad26 (ATR-ATRIP) and Rad9-Rad1-Hus1 (9-1-1) checkpoint sensor complexes are massively accumulated at telomeres and activated, due to the elimination of telomere protein Taz1. We showed that telomeres in taz1∆ cells block Chk1 phosphorylation by limiting accumulation of Crb2 (53BP1 ortholog) at telomeres, and found that forced targeting of Crb2 to telomeres leads to Chk1 phosphorylation and cell cycle arrest in taz1∆ cells, but not in wild-type cells. In order to explain this phenomenon, we investigated status of the known recruiters of Crb2, and found that 1- Rad4/Cut5 (TopBP1 ortholog) is present and activated, 2- phosphorylated H2A (γ-H2A) is also present, but 3- H4K20me2 is surprisingly missing at telomeres. Therefore, we proposed that shortage of H4K20me2 could contribute to telomere's ability to prevent Crb2-dependent cell cycle arrest. Inaddition, we determined that other subunits of fission yeast shelterin complexes, Pot1 and Ccq1, play critical roles in preventing Crb2 accumulation and Chk1 activation. Furthermore, we found that elimination of Set9, a sole enzyme responsible for generating H4K20me, me2 and me3, was able to partially rescue accumulation of Crb2 and strong cell cycle arrest phenotype found in taz1∆ ccq1∆ cells (Fig. S5D). Taken together, these observations suggested that the fission yeast shelterin complex contribute in attenuation of Chk1 activation via histone-dependent and -independent regulation of Crb2. It is also important to note that elimination of set9∆ or γ-H2A only partially abrogate checkpoint activation in response to DNA damages in fission yeast, due to the existence of CDK-dependent modulation of Cut5-Crb2 interaction that allow recruitment and activation of Crb2 at DNA damages even in the absence histone modifications. 

 We view with great interest the data presented by Audry and colleagues. Their work alerts the community that the basis of the checkpoint inhibition mechanism at telomeres is far from being fully understood. We do not view, however, that this constitutes a matter of incompatible results. 

 In fact, our results are experimentally different from the current study: 

1. We did not normalize H4K20me2 levels over H4 levels as in their Fig. 1B, instead we measured different forms of H4K20 methylation as a ratio of total input. While normalization could be useful in certain circumstances, it could also introduce unintended artefact. Thus, without evaluating their original raw data, it is nearly impossible to determine if their newly identified H4K20me2 antibody behave substantially different from antibody utilized in our previous study (Abcam ab9052). Normalization may simply reflect fewer H4 levels at telomeres and, consequently, provide an explanation to our previous finding that lessH4K20me2 and Crb2 accumulate at telomeres. 
2. We would also like to point out that the H4K20me2 antibody utilized in our study was originally validated by SL Sanders (Sanders et al. 2004, Fig. 1), and widely used (more than 20 publications to date). Using the same antibody as our study, others have since corroborated our results. ChIPseq experiments initially performed by the group of S Grewal, NIH alerted us to lower levels of this mark near telomeres (personal communication). More recently, Svensson et al. 2015, looking at genome-wide distribution of H4K20me2 (Fig S4B), independently observed reduced levels of this mark at pericentromere and subtelomere regions. Therefore, we are somewhat surprised and disappointed that this manuscript does not directly compare their newly identified antibody against the previously established H4K20me2 antibody.

In any case, a potential explanation to our seemingly contradictory results may reside on different affinities ofthe antibodies used. It may be that this new reagent is able to pick up low amounts of H4K20me2 that we were previously unable to detect. If so, this would sustain a quantitative model whereby very low levels of this chromatin modification may be unable to provide sufficient enough Crb2 to trigger a sustained checkpoint response. Nevertheless, since experimental evidences strongly suggests that Set9-dependent H4K20 methylation alone cannot provide a full explanation to the refractory nature of telomeres to Crb2 as we outlined above, we do believe that it is very important to further investigate how telomeres prevent checkpoint activation in collaboration or in parallel to regulation of chromatin status, in particular, using independent techniques that would support each other.

 We openly divulge our identity: 

 Miguel Godinho Ferreira and Toru Nakamura

We have read this submission. We believe that we have an appropriate level of expertise to state that we do not consider it to be of an acceptable scientific standard, for reasons outlined above.

# Response

**Published:** 2018-09-26

**Authors:** Kurt Runge

**Affiliations:** Cleveland Clinic Lerner Research Institute, USA

Rebuttal to Ferreira and Nakamura:

The inclusion of Drs. Ferreira and Nakamura as reviewers was a major failure of reviewer selection system for the F1000Research Preclinical Reproducibility and Robustness channel. We are directly disputing results from a paper where Drs. Ferreira and Nakamura were the senior authors.  While the editorial team tries to remove reviewers with a positive bias,they relied on software to select reviewers that included authors of the disputed publication and their former collaborators. Under this open review system, the editors and authors are supposed to agree on a set of reviewers, but reviewers were selected before we could respond.  The editorial staff did not vet these reviewers and thus allowed the inclusion of the most negatively biased reviewers possible.  As we show below, their comments distort the facts about our paper and are without merit. 

The point of our paper is that H4K20me2 is as clearly detectable in nucleosomes adjacent to telomere repeats as it is at internal loci, requiring re-evaluation of this mechanism for checkpoint suppression proposed in Carneiro et al. The review by Ferreira and Nakamura follows our discussions with them for more than one year in which we provide H4K20me2 localization and they turn the discussion to Pot1, Ccq1 and Crb2. Our paper is only about H4K20me2 localization.

H4K20me2 exclusion from nucleosomes near telomere repeats was a fundamental mechanistic conclusion of Carneiro et al. 2010.  This paper frequently states H4K20me2 is excluded from telomeres and that this exclusion is the mechanism for severing the checkpoint response.  For example, their results contain the statement:

“In contrast, H4K20me2 was undetectable at telomere in earlier wild-type or *taz1∆*cells.”

Their final model in Figure 4g diagrams this exclusion and the legend states:

            “Pot1 and Ccq1, together with the Taz1 complex, define a chromatin-privileged region that excludes H4K20me2 and prevents stable Crb2 ^53BP1^association” (spelling error corrected)

            Their penultimate paragraph in the text constituting the discussion begins with: 

            “The inability of telomeres to stably recruit Crb2 ^53BP1^is probably due to the lack of H4K20me2 epigenetic marks at chromosome ends.”  This paragraph ends with:  

            “In contrast, the absence of H4K20me2 marks regions where DNA perceived as damaged, such as chromosome ends, would not interfere with genome stability thus precluding a full checkpoint response.”

It is worth noting that Ferreira and Nakamura also cite data in their rebuttal that are not in their paper (there is no figure S5D), and overstate the claims of some of the data shown (Figure S5A shows immunofluorescence data of overexpressed Crb2 in very sick cells with multiple mutations and global changes in chromatin as a bargraph without error bars, so these data are, at best, very indirect for changes in H4K20me2 levels). They also avoid mentioning their own contradictory data that Crb2 can be detected at telomeres by ChIP with another epitope tag (presented by Dr. Nakamura at the 2013 Cold Spring Harbor Telomeres and Telomerase meeting), so the overall picture is more complex than they present.  The focus of their summary on Pot1, Ccq1, Crb2 and phosphorylated H2A is irrelevant to our paper and distracts from the main point: H4K20me2 is present in nucleosomes adjacent to the telomere repeats at levels comparable to internal loci.

We show below that the two main points raised by Ferreira and Nakamura are simply not true, misrepresent our data and contradict their own previous discussions with us.

Objection 1.  (A) Normalization to H4 levels versus total input chromatin in ChIP and (B) the absence of the original data.  

            1. A.  The normalization to input chromatin in Carneiro et al. biases the results to lower levels of nucleosomes and H4 adjacent to telomeres, and leads to the erroneous conclusion that H4K20me2 is reduced near telomeres.  Telomere repeats are bound by specific binding proteins, so chromatin fragments that include telomere repeat chromatin will have fewer histones in total.  For example, the ~300 bp of telomere repeats in wild type cells are bound by non-nucleosomal telomere-specific binding proteins (i.e. Taz1 and Pot1).  A 500 bp chromatin fragment containing the telosome will have fewer histones than an internal fragment of the same size. Normalization to input chromatin will thus yield a reduced signal for H4 and will give an artifactual decrease in all H4 modifications. Our data supporting this point are presented in the new Dataset 3 described in 1. B below.

            In contrast to normalization by input chromatin, normalization to total histone H4 at a locus allows one to assay the level of modification in the nucleosomes present, regardless of the adjacent non-nucleosomal chromatin. It allows the direct assay of the major claims in Carnerio et al. 2010 quoted above.  

            1. B.   We submitted the original data in a spreadsheet that was linked to our paper as Dataset 2, which had an associated doi in the pdf version of the paper that we proofed, but that link was not yet live active. This doi entry linked to an error webpage and was absent in the HTML version due to a processing mistake.  We are surprised that Ferreira and Nakamura state that the data were not provided when the defective hyperlink was obvious. The editorial staff would have provided the data if these reviewers asked for it.

            Our revised version contains the original Dataset 2 and has used these data to show that normalization to input chromatin gives reduced levels of histone H4 at telomeres (e.g. 1/5 to 1/9 the level of internal loci) and presents this analysis in a new third worksheet of Dataset 3. H4K20me2 signals near telomeres will therefore be low due to the artifact of normalizing non-nucleosomal chromatin fragments to input chromatin that is primarily nucleosomal.  

Objection 2.  The H4K20me2-specific antibody we used.

            We find the wording of this objection extremely odd as Dr. Toru Nakamura told us in 2012 that while the original SL Sanders antibody sold by Abcam (as ab9052) was specific and good for ChIP, this original antibody was no longer available and subsequent lots sold under the same catalog number did not work well for ChIP.  We therefore find this reversal of opinion to criticize our work puzzling, as repeating the experiment with the working anti-H4K20me2 antibody from Abcam is impossible as it no longer exists. 

            We did test the Abcam antibody sold as ab9052 in 2014 in case a newer lot showed better specificity.  However, our tests confirmed Dr. Nakamura’s statement that the newer antibody showed less reactivity with H4K20me2, and showed a different reactivity to the histones from the yeast mutants originally tested in by the Sanders lab in Greeson et al. (2008). The reactivity for the histones in the *crb2-F178Y*mutant appears to be different with the new lot of antibody and the reactivity with the pure H4K20me2 mimic is reduced (Supplemental Figure 1).  We do not know what else this antibody recognizes in the *crb2-F178Y*mutant.  In contrast, the GT282 antibody we used showed stronger selectivity and reactivity with H4K20me2 without the issues that accompany ab9052.

            We cannot comment on the Svenson et al. 2015 paper as we did not address their findings in our paper and do not know what lot of antibody they used for their experiments. We only test whether the nucleosomes adjacent to the telomere repeats are depleted in the H4K20me2 modification, and find that they are at the same level as two different internal loci.  Therefore, checkpoint suppression must occur by a mechanism distinct from H4K20me2 exclusion.

Ferreira and Nakamura include another puzzling statement after these two points.  

            “It may be that this new reagent is able to pick up low amounts of H4K20me2”. Our data show that the levels at internal loci and telomeres are the same, so any quantitative models regarding Crb2 accumulation at telomere would apply to internal loci and would not explain telomere-specific checkpoint suppression. 

In conclusion, the nucleosomes adjacent to telomere repeats have the same levels of H4K20me2 as the internal genes act1 and hip3, so some other mechanism than H4K20me2 exclusion prevents checkpoint activation at telomeres.

Kurt Runge

This rebuttal was reviewed and accepted by all authors of this paper by Audry et al.
